# Health providers´ knowledge on maternal and newborn care: implications on health systems strengthening in Vihiga County, Kenya

**DOI:** 10.11604/pamj.2020.37.73.24597

**Published:** 2020-09-18

**Authors:** Imelda Namayi, Anselimo Makokha, Elizabeth Echoka

**Affiliations:** 1 School of Public Health, Jomo Kenyatta University of Agriculture and Technology (JKUAT), Nairobi, Kenya,; 2 Department of Food Science, Jomo Kenyatta University of Agriculture and Technology (JKUAT), Nairobi, Kenya,; 3 Centre of Public Health Research (CPHR), Kenya Medical Research Institute (KEMRI), Nairobi, Kenya

**Keywords:** Maternal, newborn, health, health provider, Kenya, intrapartum, knowledge

## Abstract

**Introduction:**

pregnant women need access to skilled attendance at birth and emergency obstetric care (EmOC) to avert maternal deaths. While poor EmOC services may explain the high maternal mortality, inadequate knowledge of providers is also part of the problem. This forms the basis of this paper, in a setting where 50.2% of women deliver in a health facility but maternal mortality remains high at 531/100,000 live births, compared to the national average of 362/100,000 in Kenya.

**Methods:**

a facility based cross-sectional survey was conducted in 2018 with a set of knowledge questions extracted from the averting maternal death and disability toolkit. Providers knowledge for maternal and newborn health (MNH) was assessed by interviewing nurses on duty in the maternity units. Data were entered in Ms Access and exported to R version 3.6.2 for descriptive and logistic regression analysis. Ethical clearance was obtained from Kenya Medical Research Unit.

**Results:**

a total of 55 nurses were interviewed. Majority (71%) of the respondents were diploma nurses. The overall knowledge score for MNH among the providers was adequate with a score of (64%). Generally, the midwives and higher diploma nurses consistently scored higher than diploma nurses in all the topic areas of MNH. In the mixed linear regression, determinants of knowledge score were seen in provider-level variables.

**Conclusion:**

overall, the providers scores were higher on intrapartum and newborn care compared to scores on care for complications. We conclude that in-service training on EmOC to providers is critical to reduction of maternal mortality.

## Introduction

Available evidence indicates that while 303,000 women died in 2015 from preventable causes related to pregnancy and childbirth, sub-Saharan Africa contributed to 201,000 of these deaths. Countries in sub-Saharan Africa had very high maternal mortality rate (MMR) with estimates ranging from 999 down to 500 deaths per 100 000 live births [1]. In Kenya maternal mortality accounts for 14% of deaths among women aged 15-49, with a ratio of 362 per 100,000 live births [1,2]. Among the main causes of maternal death are postpartum hemorrhage, hypertensive disorders of pregnancy, infections and obstructed labor. For newborns, they are prematurity, intrapartum complications and infections [3]. Majority of maternal death occur during childbirth and within 24 hours after delivery which can be prevented when women deliver in a health facility where a skilled birth attendant can offer emergency obstetric care (EmOC) [4,5]. While almost 100% of births in developed regions occur with skilled birth attendants, more than half of all births in sub-Saharan Africa still occur unassisted by skilled birth attendants [6]. The high prevalence of maternal mortality in sub-Saharan Africa has been linked to poor access of skilled birth and EmOC services. Health facility delivery ensures women have access to skilled attendance. Hence increasing the rate of facility delivery has been a key strategy to improving maternal and neonatal health [7]. In Kenya, there has been minimal progress in the proportion of births attended by skilled birth attendants, as it increased marginally from 42% as reported in Kenya Demographic and Health Survey (KDHS) 2003, 44% in KDHS 2009 and 62% in the KDHS 2014 [2].

In Vihiga County, Kenya, only 50.2% of all women who gave birth were attended by skilled birth attendants at a health facility compared to 62% on a national level in 2014 [2]. Maternal mortality has been estimated to be considerable higher 531 MMR in Vihiga County compared with national estimates [2,8]. The Kenya government has rolled up many interventions and policies to enable women deliver assisted by skilled birth attendants in the health facilities. These interventions include the 'Beyond zero campaign' spearheaded by the first lady to stop preventable maternal deaths by providing fully equipped mobile clinics since 2013 and the “Linda Mama” programme administered by the National Health Insurance Fund (NHIF). In Kenya, the uptake of antenatal care services (ANC) is impressive. The KDHS findings indicate 96% of women received antenatal care from skilled provider while in Vihiga County, 97.1% women received antenatal care services [2]. However, it seems that mothers opt to deliver at home after attending the ANC and when they experience complications of pregnancy, they are brought to the health facility sometimes in very dire conditions. Assessments conducted in Malindi, Kenya, indicate ineffective management of obstetric complications is contributed by delays in making the decision to seek care when complications occur combined with delays in reaching the hospital [9]. The World Health Organization (WHO) defines EmOC as “a package of medical interventions required to treat the major obstetric complications that arise during pregnancy and childbirth” [10].

Facilities are considered EmOC facilities if they provide a series signal functions over a designated 3-month period. The seven signal functions that define a basic EmOC (BEmOC) facility are parenteral administration of antibiotics, oxytocic drugs and anticonvulsants, manual removal of the placenta, removal of retained products of conception, assisted vaginal delivery and basic neonatal care including neonatal resuscitation. Comprehensive EmOC (CEmOC) includes the seven signal functions plus performance of caesarean sections and blood transfusions. According to the UN guideline, the recommended ratio of EmOC facilities to population size is 5/500,000 [10]. The ratio of EmOC facilities to population size was (6.2/500,000) compared to the recommended 5/500,000. However, based on the strict WHO definition of basic EmOC facilities providing the six signal functions over a designated 3-month period, none of the facilities met the EmOC requirements because assisted delivery, by vacuum or forceps was not provided in any facility. Overall, though limited data exits on emergency obstetric care services in Kenya. The major gap is that the EmOC process indicators have not been integrated into the national health management information system to monitor provision of EmOC services at the facility level [11]. Assessments in low-resourced settings have shown that, in most cases, though structures are in place to support delivery of emergency obstetric care however health care providers are unable to provide all the signal functions of EmOC [12]. In Kenya, lack of knowledge and skills together with healthcare providers´ shortage is the likely reason why interventions are not delivered or delivered sub-optimally to mothers and newborns [13]. Despite the delivery of quality care being dependent on the knowledge and skills of healthcare providers, these are rarely assessed [14]. The objective of this study was to determine health providers´ knowledge on routine maternal and newborn care, treatment of complications and the factors that influence knowledge.

## Methods

**Study setting:** this study was conducted in Vihiga County in Kenya. Vihiga County is one of the 47 Counties located in the western region of Kenya and covers an area of 531 km^2^ [15]. The County has five administrative sub-Counties namely Hamisi, Emuhaya, Luanda, Sabatia and Vihiga. The total population in the County was 600,000 in 2019 [16]. The County has a total of 90 health facilities (public and private) [15]. From these 45 (50%) offer maternity services. For this study, 30 health facilities were selected on the basis of offering maternity services 24 hours a day, 7 days a week and had conducted a total of thirty (30) normal deliveries the previous three months (January - March 2018). Among them, one was the Vihiga County Referral Hospital. There were three level-4 facilities in Sabatia, Hamisi and Emuhaya sub-Counties. All the four facilities provided comprehensive EmOC while the remaining 25 facilities offered basic EmOC. The facility based cross-sectional survey was conducted in the maternity units of the 30 health facilities between April and May 2018. This study was part of a larger study reference number (KEMRI/CPHR/005/07/2015) that assessed the health systems readiness to offer emergency obstetric care (EmOC) in Vihiga County, Kenya.

**Study population:** the participants in this study were the health providers, mainly nurses on duty in the maternity units at the time of the survey. All the 30 maternity units had three or fewer nurses on duty at the time of the survey hence all nurses were invited to partake in the survey.

**Knowledge questionnaire:** a quantitative structured questionnaire was adopted from the Averting Maternal Death and Disability Emergency Obstetric and Newborn Care (EmONC) needs assessment toolkit [10]. The questionnaire determined the knowledge of health providers on routine and emergency care during pregnancy, intrapartum care, essential newborn care and care for sick newborns. A pre-test of the questionnaire was conducted in a facility outside of Vihiga County. Necessary changes to the tool were made and the final tool adopted for the study. The questionnaire was administered by an interviewer who read each question out loud in English to the participant and responded directly into the tool. Answer options were not provided to nurses; instead, nurses provided their own answers and the interviewer marked the multiple choice answers as appropriate. Because all participants were trained in English, they answered in English without a need to translate to the local language. Two interviewers conducted the survey and they were part of the research team members with extensive familiarity and training in the tool; they contributed to the design of the tool and led the piloting of the questionnaire. Data completeness and accuracy was checked on a daily basis by supervisors.

**Data analysis:** data was entered in Ms Access and exported to R version 3.6.2 for descriptive and logistic regression analysis. Based on the questions administered to the health care providers, a knowledge level score was generated. Average summary scores were then calculated for each specific question included in the topic area. Each knowledge question had multiple correct answers; that is, answers that the respondents were expected to offer spontaneously. If a correct answer was not offered, the interviewer coded the response as “not mentioned”. If a spontaneous answer did not appear as one of the multiple choices, it was not taken into consideration for scoring purposes. Respondents were scored on each question by calculating the number of correct responses mentioned out of the total possible and standardizing this to a scale of 100. A mixed-effect linear regression model was used to identify determinants of health providers´ knowledge on maternal and newborn care. The regression coefficient informs us how much the summary knowledge score is expected to increase when the independent variable increases by one, holding all other independent variables constant. In addition to the uni-variate model, we fitted three models. Model I was fitted using provider level variables, model II using facility level variables and model III was fitted on a combination of both provider and facility level variables. Akaike´s information criterion (AIC) was run to measure the model fits and complexity. For the given models fitted on the same data, the model with the smallest value of the information criterion is considered to be the best. The knowledge scores were operationally defined as follows: adequate knowledge: if the health provider answers correctly more than 50% of the questions; inadequate knowledge: if the health provider answers correctly less than 50% of the questions.

**Ethical approval:** ethical approval for this study was granted by the Kenya Medical Research Institute (SSC protocol No: KEMRI/SERU/CPHR/ 003/3277). Written informed consent to conduct this study was obtained from the director of health in Vihiga County prior to conducting the study. Written informed consent was obtained from individual participants before administering the questionnaire.

## Results

**Socio-demographic characteristics:** in this study a total of 55 respondents completed the survey with a response rate of 99%. Out of those 49 (89%) were females. The age of the respondents ranged from 20 years to 52 years where 86% of the health providers were aged between 20-40 years. The median age of the respondents was 30 [IQR: 22-38] years. The majority 39 (71%) were diploma nurses and 10 (18%) were midwives. Majority of the respondents were working in the health centre 40 (73%). Working experience of the respondents ranges from 1 to 35 years; median 5 years [IQR: 3-12] years. The monthly average deliveries conducted by health providers was between 7 and 10 deliveries (Table 1).

**Table 1:** socio-demographic characteristic of respondents

Demographic factor	Category	Frequency (N=55)	Percentage (%)
Respondents age (in years)	20-30	38	69
	31-40	9	17
	41-50	5	9
	Above 51	3	5
Sex	Male	6	11
	Female	49	89
Education status	Midwife	10	18
	Nurse (higher diploma)	6	11
	Nurse (diploma)	39	71
Type of health facility	County referral hospital	2	3
	Sub county hospital	13	24
	Health centre	40	73
Working experience (in years )	1-5	26	47
	6-10	11	20
	11-15	5	10
	16-20	4	7
	21-25	4	7
	26-30	4	7
	31-35	1	2
Deliveries attended the previous month	1-5	35	63
	6-10	13	24
	11-15	7	13

**Figure 1:**
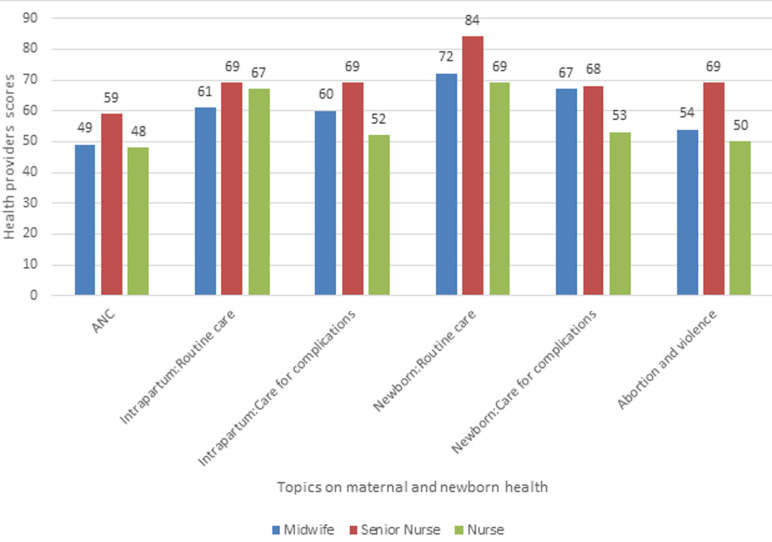
health providers’ summary knowledge score on topics of maternal and newborn health care

**Knowledge of care during pregnancy, intrapartum, abortion and violence, and newborn care:** knowledge scores were aggregated by cadre and presented in Table 2. The questions on ante-natal care were two. The health providers scored 62% on the questions on primary aspects of focused antenatal care and 36% on the question on which women require a special care plan. The health care providers scored higher on questions related to routine intrapartum and new born care than they did on care for maternal and newborn complications. Scores were highest (81%) for questions regarding observations made when monitoring a woman in labour, immediate newborn care (71%) and steps of Active Management of Third Stage of Labour (AMTSL) (70%). The scores were lowest on signs and symptoms of newborn infection (47%). The overall knowledge score for maternal and newborn health among midwives, higher diploma nurses and diploma nurses was 63, 71 and 58 respectively (Table 2). The knowledge scores were higher among providers working in high-volume facilities indicating greater exposure to mothers and newborn with complications. In general, midwives and nurses with higher diploma consistently scored higher than diploma nurses in all topic areas of maternal and newborn health care (Figure 1).

**Table 2:** provider knowledge scores (out of 100) related to a set of questions on antenatal care, intrapartum, newborn, abortion care and violence by health worker cadre in Vihiga County, Kenya

	Midwife (diploma)	Nurse (higher diploma)	Nurse (diploma)	Total
	(n=10)	(n=6)	(n=39)	
**Antenatal care**				
Primary aspects of focused antenatal care (n=6)	70	70	59	62
Women who require a special care plan (n=8)	27	48	36	36
**Intrapartum**				
**Routine care**				
Identifying a woman in labor (n=4)	65	71	68	68
Observations to monitor a woman in labor (n=9)	81	80	82	81
Steps of active management of the third stage of labor(n=4)	58	79	72	70
**Care for complications**				
Management principles for women with heavy bleeding after delivery (n=8)	63	69	50	54
Management principles for women with retained placenta (n=10)	53	66	47	50
**Newborn**				
**Routine care**				
Immediate newborn care (n=11)	72	84	69	71
**Care for complications**				
Signs and symptoms of newborn infection and or sepsis (n=7)	54	57	44	47
Care for low birth weight newborn ( < 2.5kgs) (n=5)	64	70	51	56
Diagnose of birth asphyxia (n=4)	68	67	62	63
Sequential steps of neonatal resuscitation (n=7)	61	68	50	54
Were the steps mentioned in sequential order (n=1)	90	100	92	93
Steps of newborn resuscitating with bag and mask (n=5)	90	67	54	62
**Abortion care and violence**				
Immediate complications of an unsafe abortion (n=5)	46	80	56	57
Steps to treat women with unsafe abortion (n=9)	54	69	47	51
Information to provide to clients treated for unsafe abortion (n=6)	62	56	48	52
Steps to take when treating a woman who is a victim of rape (n=8)	55	71	48	52
Total knowledge (score out of 100)	63	71	58	64

**Determinants of health providers´ knowledge on maternal and newborn care:** the determinants of health providers´ knowledge on maternal and newborn care are presented in Table 3. Model III provided the best fit with the lowest AIC (57.55). Model III was fitted on a combination of both provider and facility level variables (Table 3). The positive coefficients of knowledge score were seen in variables such as: provider cadre, number of deliveries attended in the previous month (except 6 to 10 level), years of experience since professional qualification (except 16 to 20 level), number of trainings received on maternal and newborn services and where providers received training in newborn resuscitation. Negative coefficients of the knowledge score was observed across all the facility levels variables and on the number of maternal and newborn services delivered in the previous three months (Table 3).

**Table 3:** multivariable mixed-effects linear regression analysis to identify the provider and facility-level determinants of clinical knowledge on maternal and newborn care in Vihiga County, Kenya

Variables	Univariate model		Model I		Model II		Model III	
	β Coef.	P-value	β Coef.	P-value	β Coef.	P-value	β Coef.	P-value
**Provider-level variables**								
Provider cadre								
Midwife	1							
Senior nurse	16.7188	0.992	35.66	0.9956			189.5	0.996
Nurse	-0.1542	0.841	0.45	0.7254			74.83	0.996
**Number of deliveries attend in the previous month**								
1 to 5	1							
6 to 10	-0.3102	0.6466	-0.3745	0.6879			-54.8	0.996
11 to 15	16.7859	0.991	36.23	0.9955			153.2	0.997
More than 15	-	-	-	-			-	-
**Years of experience since professional qualification**								
1 to 5	1							
6 to 10	2.303	0.0398	2.566	0.0533			133.5	0.996
11 to 15	1.386	0.242	1.375	0.5104			113.2	0.996
16 to 20	1.099	0.3677	-17.1	0.9964			-19.26	0.999
21 to 25	18.57	0.9955	20.45	0.998			40.51	0.999
26 to 30	18.57	0.9955	19.69	0.9977			208.6	0.996
31 to 35	18.57	0.9977	20.31	0.9991			99.96	0.999
Number of maternal and newborn services delivered in the previous three months	-0.1296	0.345	-0.0384	0.8279			-0.1438	0.671
Number of trainings received on maternal and newborn services	-0.0925	0.591	0.00705	0.9746			0.3248	0.574
**Training in newborn resuscitation**								
In-service	1							
Pre-service	-1.424	0.105	0.5615	0.7299			73.51	0.996
Both in and Pre- service	-0.7621	0.41	0.5464	0.7502			91.47	0.996
Other	-	-	-	-			-	-
**Facility-level variables**								
**Facility managing authority**								
Public	-15.18	0.995			-15.54	0.995	-119.6	0.999
Private owned	-16.57	0.994			-17.02	0.994	-320	0.999
Faith Based Owned	-16.97	0.994			-17.34	0.994	-175.4	0.998
**Level of Facility**								
County referral hospital	1							
Sub-County hospital	-14.76	0.992			-15.54	0.995	337	0.997
Health Centre	-14.68	0.992			-15.08	0.995	431.3	0.997
**Model Fitness**								
AIC			72.93		70.24		57.55	

Model I was fitted on provider level variables, Model II on facility level variables and Model III on a combination of both provider and facility level variables.

## Discussion

The study aimed to determine health providers´ knowledge on routine maternal and newborn care, treatment of complications and the factors that influence knowledge in Vihiga County, Kenya. Majority of maternal death occur during childbirth and within 24 hours after delivery which can be preventable when women deliver in the health facility where a skilled birth attendant can offer EmOC [5]. Generally, the knowledge score for maternal and newborn health was adequate among all the respondents. The midwives and higher diploma nurses consistently scored higher than diploma nurses in all the topic areas of maternal and newborn health care. Additionally, all the providers scored higher on routine aspects of intrapartum and newborn care compared to aspects on intrapartum and newborn complications. The knowledge scores were higher among providers working in high-volume facilities indicating greater exposure to mothers and newborns with complications. Considering the important role of nurses in skilled birth and EmOC and the regular absence of obstetricians in our setting, it is critical that nurses have adequate knowledge to provide quality care [6,7]. Available evidence conducted in sub-Saharan African countries and Asian countries on determining the provider knowledge before and after in -service EmOC training found that the training was associated with improved knowledge of health providers.

The study concluded EmOC training as an intervention to reduce maternal and neonatal mortality [8]. Previous studies from Kenya on providers´ knowledge on routine newborn and care for complication revealed that knowledge of neonatal resuscitation and checking signs of sick newborns was poor. Also, the nurses who had received in-service training scored better than those who had not [17-19]. The findings coincide with this study where the provider knowledge on care for newborn complications is inadequate. This is of concern because the ability to manage obstetric complications is very critical in reducing maternal and newborn mortality. In Uganda, a study conducted among health provider on knowledge on prenatal and newborn care, being a general nurse was associated with having adequate knowledge while type of health facility was not associated with having adequate knowledge. Although the study had a large sample size of 183 respondents, the findings were similar with this study where facility level variables were not significantly associated with having adequate knowledge [20].

Knowledge assessments, like the one conducted in this study, may be a useful complement to Kenya service delivery indicator surveys in identifying existing gaps and especially the topics for the most needed trainings. This will support the alignment of the available resources to where the greatest need is in reducing maternal and neonatal mortality. Our study has both strengths and limitations. Through the face to face interview with the respondents, we were able to substantiate or confirm the information obtained. Additionally, the coverage of the entire Vihiga County and interviewing providers from all the facilities offering maternity services was a strength. The major study limitation was that the assessment was based on respondents´ reports rather than direct observation, which might have led to some reporting error. Most of the questions had multiple correct answers that required spontaneous responses; this may have biased scores towards the end. The limitation was handled by having a clear analysis plan for the results key variables of interest in the tool.

## Conclusion

Overall, the knowledge score for maternal and newborn health was adequate among all the respondents. Health providers scores were higher on routine intrapartum and newborn care compared to scores on care for complications. There is need for regular in-service training to health providers and incorporate the trainings in the County policy documents addressing reduction of maternal mortality.

### What is known about this topic

- Assessments in low-resourced settings have shown that, in many cases, structures are put in place to support delivery of emergency obstetric care (EmOC) but health care providers are unable to provide all the signal functions of EmOC;
- Training of health care providers offering maternity services on emergency obstetric care, may help improve competency of the providers in offering quality maternal and newborn care;
- Approaches to assessing knowledge have increasingly emphasised the use of vignettes, which may provide more insight compared to use of multiple choice questionnaires, while still remaining practice and standardisable.

### What this study adds

- Gaps in health care provider knowledge may be a key priority area for quality improvement in settings with high maternal mortalities; especially knowledge on diagnosis, management of infections in newborn and the sequential steps in newborn resuscitation;
- This study highlights the need to consider the knowledge of nurses as a dimension of quality of care during facility assessments;
- Attention needs to be paid to improving nursing competency in use of partograph when monitoring a woman in labour, management of infections in newborns and sequential steps of newborn resuscitation.
